# Theoretical modeling for radiofrequency ablation: state-of-the-art and challenges for the future

**DOI:** 10.1186/1475-925X-5-24

**Published:** 2006-04-18

**Authors:** Enrique J Berjano

**Affiliations:** 1 Center for Research and Innovation on Bioengineering, Valencia Polytechnic University, Camino de Vera s/n, 46022 Valencia, Spain

## Abstract

Radiofrequency ablation is an interventional technique that in recent years has come to be employed in very different medical fields, such as the elimination of cardiac arrhythmias or the destruction of tumors in different locations. In order to investigate and develop new techniques, and also to improve those currently employed, theoretical models and computer simulations are a powerful tool since they provide vital information on the electrical and thermal behavior of ablation rapidly and at low cost. In the future they could even help to plan individual treatment for each patient. This review analyzes the state-of-the-art in theoretical modeling as applied to the study of radiofrequency ablation techniques. Firstly, it describes the most important issues involved in this methodology, including the experimental validation. Secondly, it points out the present limitations, especially those related to the lack of an accurate characterization of the biological tissues. After analyzing the current and future benefits of this technique it finally suggests future lines and trends in the research of this area.

## Introduction

Radiofrequency (RF) techniques have been used to heat biological tissues for many years. However, in recent years its use for new medical applications has expanded enormously [1]. To illustrate this, although the term "radiofrequency ablation" is relatively new, the number of papers published per year on this topic has risen from 19 in 1990 to 828 in 2005.

Radiofrequency ablation (RFA) is a (more or less invasive) interventional technique that in recent years has come to be employed in very different medical fields, such as the elimination of cardiac arrhythmias (using catheter or intraoperatively) [2], or the destruction of tumors in different locations (liver [3], kidney [4], lung [5], bone [6], prostate [7], and breast [8]). The procedure is based on RF (≈500 kHz) electrical currents passing through biological tissue and so achieving the controlled heating of the zone with the highest power density (maximal SAR, Specific Absorption Rate).

From a procedural point of view, RFA generally uses a pair of electrodes: an active electrode with a small surface area that is placed on the target zone, and a larger dispersive electrode to close the electrical circuit. On occasions, bipolar ablation is conducted with two active electrodes. In addition, using the same biophysical foundation described for RF ablation, other surgical fields use it to treat other pathologies, e.g. the correction of refractive errors in ophthalmology [9], the thermal remodeling of oral cavity tissue to cure sleep obstructive apnea [10], the intervention to minimize gastric reflux by applying RF energy to the gastroesophageal junction [11], and the therapeutic heating of the articular cartilage [12].

In order to investigate and develop new techniques, and also to improve those currently employed, research can call upon clinical and experimental (ex vivo and/or in vivo) studies, phantoms and theoretical models. The latter are a powerful tool in this type of investigation, since they provide vital information on the electrical and thermal behavior of ablation rapidly and at low cost, quantifying the effect of various extrinsic and intrinsic factors on the electrical current and temperature distribution. Consequently, they facilitate the assessment of the feasibility of new electrode geometries, and new protocols for delivering electrical power. Despite the fact that several research groups are currently using computer modeling to investigate RF ablation procedures, to date no review articles have been published on this topic. A previous review by Strohbehn and Roemer [13] dealt with computer simulations for hyperthermic treatments. Since this topic is related to RF ablation, some of the data that it provides could be useful for RF ablation modeling. Finally, various papers have briefly reviewed different methodological issues related to RF ablation modeling [14,15].

### Modeled ablation procedures

To date, theoretical modeling applied to the study of RF heating techniques have mainly focused on relatively new therapies, such as cardiac ablation [14], cancer ablation [16], and cornea heating [17]. However, other groups have used previous theoretical models to study different aspects of the RF heating phenomenon. For instance, Overmyer et al [18] and Kim et al [19] developed three-dimensional models to calculate current density and temperature distributions in the tissue under a circular electrode. Likewise, Wiley and Webster [20] studied analytically the current density distribution in the tissue under circular dispersive electrodes. These studies dealt with circular dispersive electrodes in order to clarify the origin of the perimetrical burning of the skin. In contrast, dealing with the theoretical modeling of active electrodes during RF heating, Erez and Shizter [21] used a one-dimensional model to study the effect of different factors on the temperature distribution in generic biological tissue. All these models assumed numerous simplifications (such as the homogeneity and isotropy of the tissue, blood perfusion rate unaffected by the heating process, no boiling of tissue during heating) which are considered in most current models.

More recently, other researchers have developed models for RF cardiac ablation. The first was proposed by Haines and Watson [22] and was a one-dimensional model based on a spherical electrode, which, despite its simplicity, gave valuable insight into the mechanism of RF ablation. However, it ignored important factors such as blood flow and the temperature dependence of the electrical conductivity of cardiac tissue. These issues were taken into account by Labonté [23,24], who developed a 2-dimensional model (based on axial symmetry) and validated it by means of thermograph measurements using a phantom of tissue-equivalent material. Simultaneously, Vahid Shahidi and Savard [25] and Kaouk et al. [26] proposed a new model (three-dimensional but with two-dimensional potential) incorporating fragments of blood, myocardium and torso. In 1995, the most prolific group in theoretical modeling of RF ablation directed by Prof. John G. Webster (University of Wisconsin-Madison) presented the first three-dimensional RF cardiac ablation model [27]. This group later became the leader in the modeling for RF cardiac [14,28-33] and hepatic [16,34-39] ablation. Previously, Curley and Hamilton [40] had developed a one-dimensional model for RF hepatic ablation incorporating the simultaneous infusion of heated saline into the tissue.

During the last 10 years, another two groups became interested in theoretical RF ablation modeling. The Duke University group [41-46] developed interesting three-dimensional RF cardiac ablation models to assess the effect of different factors on the temperature distributions in the tissue. This group focused mainly on increasing the realism of the modeling of blood flow [44], and conducted excellent experiments to validate their models [44,45,47]. The Valencia Polytechnic University group began their modeling studies on RF heating of the cornea [17,48,49], and later developed models for RF cardiac ablation, specifically studying the problem of thermal injury in the esophagus during RF ablation of the left atrium [50-53]. In addition, other notable modeling studies have been developed for RF hepatic ablation [54-58], RF ablation of breast cancer [59], and RF cardiac ablation, incorporating both the heat convection due to blood circulation and the irrigation of saline on the epicardium [60].

## Description of methodology

This section deals with the main steps in the building and use of a theoretical model in studies on RF heating. These steps are basically: 1) observation and simplification of the physical situation, 2) arrangement of the mathematical equations which rule the thermal and electrical phenomena, 3) determination of the boundary conditions, both electrical and thermal, 4) obtaining the physical characteristics (thermal and electrical) of the biological tissues and other materials included in the model, 5) choosing a numerical method in order to computationally or analytically achieve a solution, and 6) conducting the post-processing of the computed results. Since most models are based on the Finite Element Method (FEM), the following steps have been tailored to this methodology.

### 1) Simplifying the actual physical situation

**Figure 1:**
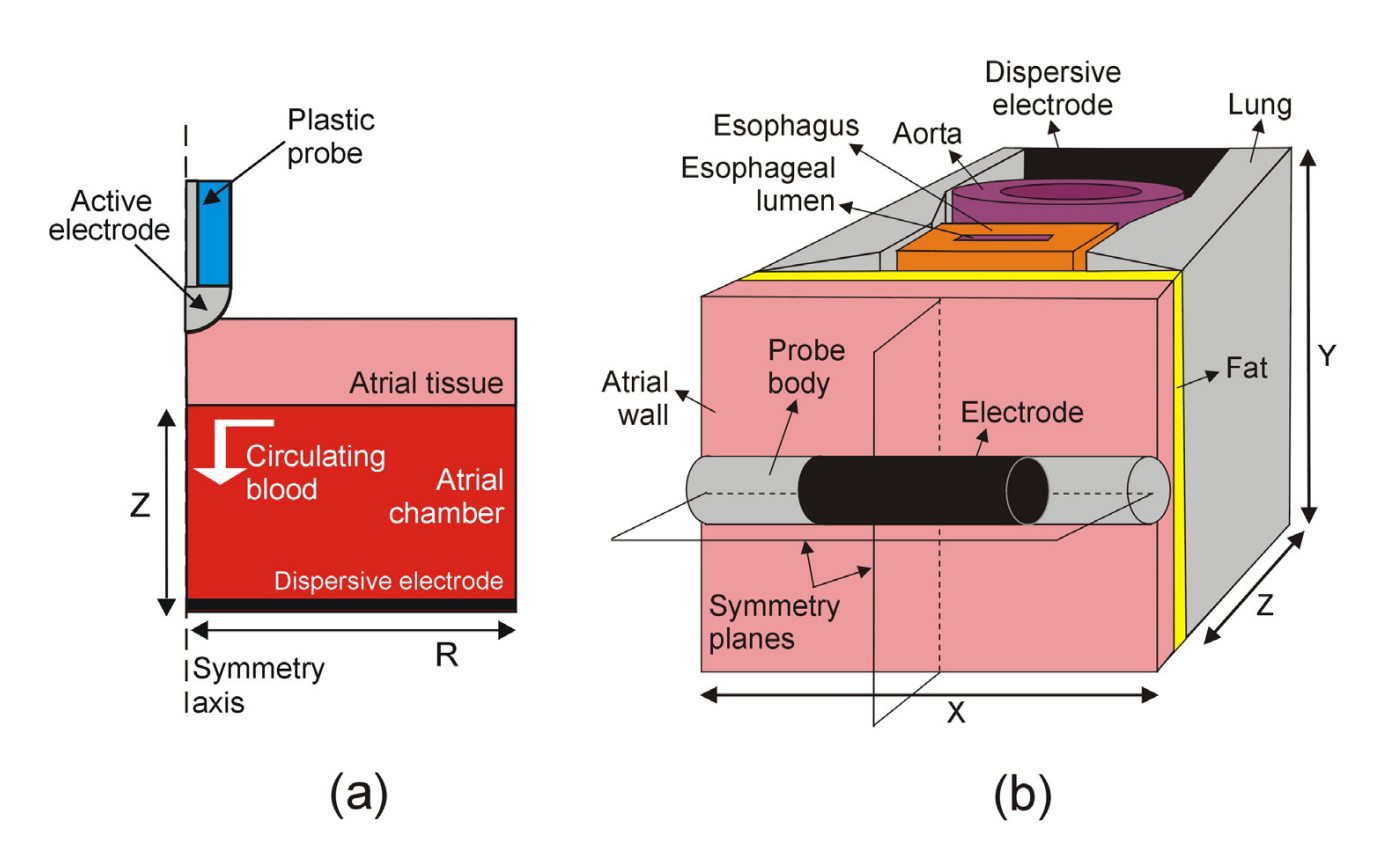
Two examples of simplifying the actual physical situation during RF cardiac ablation. (a) The axial symmetry allows a three-dimensional problem to be reduced to a two-dimensional problem in a theoretical model that includes an active electrode placed perpendicular to a fragment of cardiac tissue [52]. (b) The region under study has two symmetry planes, hence only one quadrant of the whole model can be considered for computational analysis in a theoretical model including fragments of different tissues [50,51,53]. Fig. 2 shows the only quadrant considered in the model.

The theaters in which RF ablation is performed are often too elaborate and it is therefore absolutely necessary to begin by studying this problem in detail and then to carry out appropriate simplifications. These could include, for instance, looking for planes or axes of symmetry (see Fig. 1), which would allow a three-dimensional model to be simplified into a two-dimensional model (axial symmetry case) [14,17,23,24,30,33,35,41,48,49,52], or even better, condense the physical problem to a single dimension [22,40]. Likewise, it is standard practice to consider only the most significant tissues, i.e. to overlook microscopic structures such as epithelia, basal laminas, glands, nerves, etc. In fact, only the values of electrical and thermal characteristics for whole tissues are usually available in the literature. Consequently, theoretical models only consider macro-fragments of tissue, e.g. cardiac, adipose, blood and connective.

### 2) Equations governing the phenomena

The second step consists of setting the equations governing the physical phenomenon of electrical-thermal heating. All the models of RF heating are based on a time domain analysis of a coupled electric-thermal problem. The spatial distribution of temperature in the tissues is obtained by solving the so-called Bio-heat equation [61]:



where *ρ *is the mass density (kg/m^3^), *c *is the specific heat (J/Kg·K), *k *is the thermal conductivity (W/m·K), *T *is the temperature (°C), *q *is the heat source (W/m^3^), *Q*_*p *_is the perfusion heat loss (W/m^3^), and *Q*_*m *_is the metabolic heat generation (W/m^3^). This last term is always ignored since it has been shown to be insignificant for ablation [24]. Likewise, the situation is often simplified by ignoring *Q*_*p *_as it is negligible in some cases of RF heating, such as non vascular tissues [17,48,49]. On the other hand, *Q*_*p *_is always considered in cases of tissues with a high degree of perfusion, such as liver [16,34,36,37,40,54,55]. The case of in vitro ablation of excised liver could be an exception [35]. Regarding RF cardiac ablation, *Q*_*p *_is incorporated in some models [25,27,28,62], but is generally ignored [14,23,24,31,32,43,44,52,63] since its effect is negligible for cardiac ablation [22]. This term is mathematically expressed as:

*Q*_*p *_= *ω*_*b*_·*c*_*b*_·(*T *- *T*_*b*_)     (2)

where *ω*_*b *_is the blood perfusion per unit volume (kg/m^3^·s), *c*_*b*_, is the specific heat of blood (J/Kg·K), and *T*_*b *_is the blood temperature (°C). In general, *ω*_*b *_has been assumed as uniform throughout the tissue. However, in a few studies its value was increased with heating time because of vasodilation and capillary recruitment [64] or annulled to model the cessation of local blood flow due to tissue necrosis [54].

At the frequencies employed in RF ablation (300 kHz – 1 MHz) and within the area of interest (it is known that the electrical power is deposited within a small radius around the active electrode), the tissues can be considered purely resistive, because the displacement currents are negligible. For this reason, a quasi-static approach is usually employed to resolve the electrical problem [65,66]. Then, the distributed heat source *q *(Joule loss) is given by

*q *= ***J·E ***    (3)

where ***J ***is the current density (A/m^2^), and ***E ***is the electric field intensity (V/m). The values of these two vectors are evaluated using Laplace's equation:

∇ · *σ *∇*V *= 0     (4)

where *V *is the voltage (V) and *σ *is the electrical conductivity (S/m). By using the quasi-static approach, the values of "direct-current" (DC) voltage calculated from the model correspond with the root mean squared (r.m.s.) value of the RF voltage actually employed.

The equations (l)-(4) give the solution of an electrical-thermal coupled problem which generally represents adequately the RF ablation of biological tissues. However, some models have incorporated additional terms into the Bio-heat equation or have employed extra equations which describe other physical phenomena. For instance, in order to improve the prediction of the temperature in the circulating blood during RF cardiac ablation, the Mass Equation and Momentum Equation have been employed to solve a thermal-flow coupled problem [44,63]; other models have used fluid dynamics theory to derive a velocity field for the saline flowing out of the electrode holes during RF epicardial cardiac ablation using irrigated electrodes [60]; and other studies have modeled RF hepatic ablation using irrigated electrodes in which heated saline is simultaneously infused into the tissue [40]. In this last case, the tissue can be considered as a porous medium into which saline is infused, and hence additional equations should be employed (e.g. Darcy's Law) [67]. As opposed to this complexity in the formulation, the modeling of internally cooled RF ablation electrodes (dual-channel probes with an electrode at the tip, in which cooling fluids are continuously delivered through one channel and removed through another) have been roughly approximated by means of a single temperature boundary condition at the electrode surface (value fixed to the same value as the cooled fluid) [41,68].

### 3) Initial and boundary conditions

Once the thermal and electrical equations have been stated, it is necessary to set the boundary conditions, both thermal and electrical. RF ablation is typically performed using a constant-voltage. In this case, the electrical boundary conditions can be of two types: null current (Neumann boundary condition) at the symmetry axis [17,48,49,52,68] and planes [50,51,53], at points remote from the heating zone [43,45], at the air-tissue interface [17,48,49,52]; and fixed voltage on the electrodes (Dirichlet boundary condition), in particular 0 V at the dispersive electrode, and ≠0 V at the active electrode [17,23-25,41,44,48,49,52,54,55,59,62]. Conversely, in the case of a constant-current ablation, a value for current ≠0 A is fixed at a point on the active electrode, and the same value, but negative, is fixed at a point on the dispersive electrode (null voltage has to be fixed on the dispersive electrode as well). Moreover, the usual practice is to model a constant-temperature RF ablation, in which the delivered electrical power is modulated by the RF generator to maintain at a preset value the temperature of a sensor located in the electrode. In this case, a boundary condition of fixed voltage on the active electrode is used, but its value is adjusted to keep the sensor temperature constant [14,16,29,31,32,34,36,37,42,43,45,46,50,51,53]. This procedure involves manual adjustment based on a costly trial-and-error method. In this respect, a closed loop control joined to the theoretical model allows not only a temperature controlled RF ablation to be modeled with minimal user input, but also gives results comparable to clinical devices that use this type of control [33,69]. Other studies also modeled constant-power ablation [14,32,42,43,70], which is a clinically employed procedure. Finally, some models included ablation with controlled impedance mode, i.e. maintaining the impedance value lower or higher than a preset value [35].

Thermal conditions can be of two types: 1) null thermal flux (Neumann boundary condition), for instance, at the symmetry axis and plane [17,52]; 2) constant temperature (Dirichlet boundary condition) for instance, at points distant from the heated zone [17,24,27,42,48,49,71], at the same active electrode used to model an internally cooled electrode [41,68], and 3) thermal convection (forced or free) modeled by means of a thermal transfer coefficient, e.g. at the air-tissue interface [17,25,48,49,52], and the endothelium-blood interface in cardiac chambers [24,25,27,45].

In addition, a value for the initial temperature has to be considered for transient thermal analyses. This value is frequently equal to those chosen in the experiments with which the computer simulations will be later compared. Almost all the studies modeling clinical RF ablations considered normothermic values of 37°C [25,27,36,37,41,42,44-46,71,72], 36°C [52] or 35°C [17]. Occasionally, hypothermia values (32°C) were considered to model this condition during a surgical RF cardiac ablation [50,51]. Finally, other models in which the computer results were compared to ex vivo experiments, the value of initial temperature corresponded with an ambient temperature of 20–25°C [35,48,49,59].

### 4) Physical characteristics of biological tissues

In order to build the complete theoretical model, the value of four physical characteristics have to be set for all the material of the model: mass density (*ρ*), specific heat (*c*), thermal conductivity (*k*), and electrical conductivity (*σ*). All these values are usually taken from the scientific literature [73-76], specifically from previous experimental studies of measurements on ex vivo and/or in vivo biological tissues, and considered in the same conditions as those used during RF ablation, i.e. at ≈500 kHz for the *σ*, and at the appropriate temperature. If no previous data are available for a certain tissue, it is possible to consider the characteristics of a histologically comparable tissue.

All the characteristics are normally considered to be isotropic. In this respect, it has been experimentally demonstrated that the anisotropy of *σ *at RF frequencies is not significant [77,78]. On the other hand, although no experimental data are available on the anisotropy of *k*, some modeling studies have considered the impact of a possible anisotropy on this parameter by means of computer simulations [48,79].

An important issue that has received little attention to date is the relationship between tissue characteristics and temperature. Although some RF ablation models did not consider any relationship [27,35,44,60], most RF ablation models considered a temperature-dependent change in *σ *using a temperature coefficient of +2%/°C [14,23-25,28,33,43,45,52,59,62,80] or a polynomial relation derived from NaCl solutions [54,55]. However, this value was experimentally obtained from measurements made below 42°C [81]. For this reason, Pop et al [82] have recently measured the change of *σ *(at RF frequencies) during heating and have found that this phenomenon follows an Arrhenius model which allows the modeling of irreversible changes in *σ*. Likewise, the parameter *k *has been traditionally considered constant. Only a few models incorporated a linear relation with temperature [17,25,28,50,51,53,62]. Recently, Bhattacharya and Mahajan [83] have experimentally observed a linear relation below a threshold temperature (variable for different tissues). Over this threshold, irreversible changes occur, involving a sort of hysteresis in the relation *k*-T.

On the other hand, many RF ablation procedures involve a temperature of nearly 100°C. In values of this order, it is known that non-linear phenomena occur, such as desiccation and vaporization (bubble formation) [84]. Since gas formation and desiccation are associated with an increment of the electrical impedance, Haemmerich et al [35] modeled this phenomenon using a coefficient of value +2%/°C below 100°C, and assumed a rapid drop in *σ *by a factor of 10000 between 100 and 102°C (applying additionally a latent heat associated with water vaporization). However, this approximation does not take into account the irreversible behavior of *σ*, and hence the results do not match the real situation, which is without doubt much more complex. For this reason, other modeling studies decided to end the computer simulation when the maximal temperature in the tissue reached 100°C [17,52,60], or to modulate the RF energy in order to maintain the maximum temperature at 95–100°C [23,24,27,34,36,41,43,69]. This is a more reasonable choice, which involves the loss of information on the phenomenon outside this limit. However, the objective of the modeling study is usually confined to knowing whether this temperature limit is reached during heating [17].

More recently, some interesting attempts have been made to quantify the relationship between temperature and specific heat [85], and to measure the characteristics of biological tissues under different physiological conditions [86] and pathological states [87].

### 5) Numerical method and computer-based solution

To obtain the solution of the equations governing the physical phenomena during RF ablation it is necessary to chose a calculation method. Sometimes, the geometry of the model (e.g. in one dimensional models) is simple enough, and these equations can be solved by analytic methods [20,22]. However, most models present a complex geometry (sometime based on a very realistic anatomy), with regions of different characteristics, and a numerical method has to be employed, such as the Finite Differences Method (FDM) or the FEM [13]. In the case of a numerical method, the solution is obtained by means of a computer. The FDM generally has less computation requirements (memory and time) and consequently has been employed for problems presenting a simple geometry [40,88,89]. However, at the same time, FDM allowed to implement problems involving a mathematical formulation more complex than those found in FEM studies [40,89-91].

Concerning the use of the FEM, although some groups have developed their own software [23,24], most have employed commercially available software. For instance, most RF ablation models have used ANSYS [17,28,30,41-45,48,53,62,92] since it is able to perform electrical-thermal coupled field analysis with temperature-dependent properties. However, the main disadvantage of ANSYS is its cost. Other models have combined different programs for each step of the procedure. Firstly, a pre-processing program is necessary to create the geometric model and to assign material properties as well as the boundary conditions to each region [28]. Several programs have been employed for this such as INGRID [27], MSC/PATRAN [25,28,29,35,80], NETGEN [60], and IDEAS [63]. Secondly, once the model has been built, a solver program such as TOPAZ3D [27], COSMOS [27,70,71], ABAQUS [25,28,35,63,69,80], and GMRES [60] can be used to obtain the solution. Finally, post-processing programs such as TAURUS [27] and ABAQUS/POST [29] have been employed for displaying the results. The combination of PATRAN-ABAQUS-ABAQUS/POST for preprocessing-solver-post processing, respectively, has also been extensively employed [14,16,32,34,36,37].

Some groups have recently employed FEMLAB (COMSOL in the present version) in their modeling studies [54,55,59]. This program, like ANSYS, provides all the elements necessary to build the model, to solve the problem and post-process the results. Moreover, its basic version allows arbitrarily defined equations to be introduced, and coupled problems to be solved using these same equations. This could become an important advantage in the future, since it would allow complex coupled problems (flow-thermal-electrical) and the intricate relationships between temperature, tissue damage and boundary conditions to be determined. In fact, it has produced hybrid models using FEMLAB and MATLAB to set up interesting relationships between computed temperature, tissue damage and resultant cessation in local blood flow [54].

**Figure 2:**
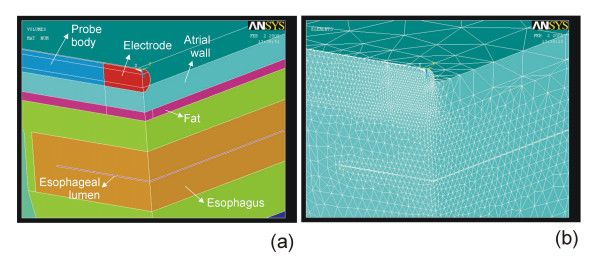
Example of meshing using tetrahedral elements. (a) Zoom of the only quadrant considered in the three-dimensional model of the Fig. 1b. (b) Meshing using tetrahedral elements. The mesh size is smaller where the gradient of electric field and current density are maximum, i.e. where the heating occurs. The optimum value of this parameter is calculated by convergence tests.

Most FEM programs have numerous advantages for building, solving and post-processing models (such as a user-friendly graphical interface, ease of complex model generation), however three key issues have to be take into account in order to obtain accurate solutions. Two of them are related to the discretization processes carried out during FEM: 1) spatial discretization of the model region by creating a mesh (usually triangular elements for two-dimensional models and tetrahedral elements for three-dimensional models) (see Fig. 2), and 2) time discretization during transient analysis by establishing time steps. Finally, the models for RF ablation always include a single fraction of the tissues included in the real situation, in particular from the area in which the heating occurs, i.e. where the gradient of electric field and current density are maximum. This implies that the outer dimensions of the theoretical model are arbitrarily set (see Z and R in Fig. 1a, and X, Y, and Z in Fig. 1b). Consequently, an essential step in theoretical modeling is to determine the optimum values for mesh size, time step, and outer dimensions. This can be achieved by means of sensitivity and convergence tests.

Regarding the outer dimensions of the model, the correct choice is a compromise between a model that is large enough to yield a valid solution and small enough to require reasonable computing time and memory [24]. A sensitivity analysis allows the appropriate outer dimensions to be obtained [93]. The procedure is as follows: initially, values of outer dimensions are arbitrarily chosen. Then, the model is tested (by means of several computer simulations using typical conditions of time, delivered power, etc) by increasing the value of the outer dimensions in each simulation by gradual stages. It is also necessary to chose a control parameter for this sensitivity analysis such as the total impedance between electrodes [24], or the maximal temperature achieved in the tissue after a time [49,52]. When the difference between the value of the control parameter in a simulation and its value in the previous simulation is less than the threshold (previously chosen), the former values of the outer dimensions can be considered as appropriate. The threshold value can be an absolute value (depending on the chosen control parameter) or a relative value expressed as a percentage of the former value (typically 0.5%) [52].

Likewise, the optimum mesh size and time step are determined by a similar procedure called a "convergence test" which is described in detail in [14]. In this case, the control parameters employed were the temperature at the electrode tip [14], the temperature in reference nodes (arbitrarily chosen) [44], or the maximal temperature in the tissue [48,49,52]. The threshold values chosen in the studies were 0.1°C [14], 0.5°C [48,49], or 0.5% [43,44,52].

The determination of the optimum values of mesh size, time step and outer dimensions is actually a combined process, since any sensitivity and convergence test for determining one parameter is implicitly employing a value used by the others. For this reason, it seems appropriate to conduct a more or less iterative process, for instance, to consider initially a tentative spatial and temporal solution (e.g. a small grid size in the heating zone, usually the active electrode-tissue interface, and a small step time, 25–50 ms). Then, a computer analysis is conducted to determine the appropriate values of the outer dimensions. Finally, once these values have been obtained, convergence tests are performed to determine adequate spatial and temporal discretization [52].

### 6) Post-processing: output variables and assessing lesions

**Figure 3:**
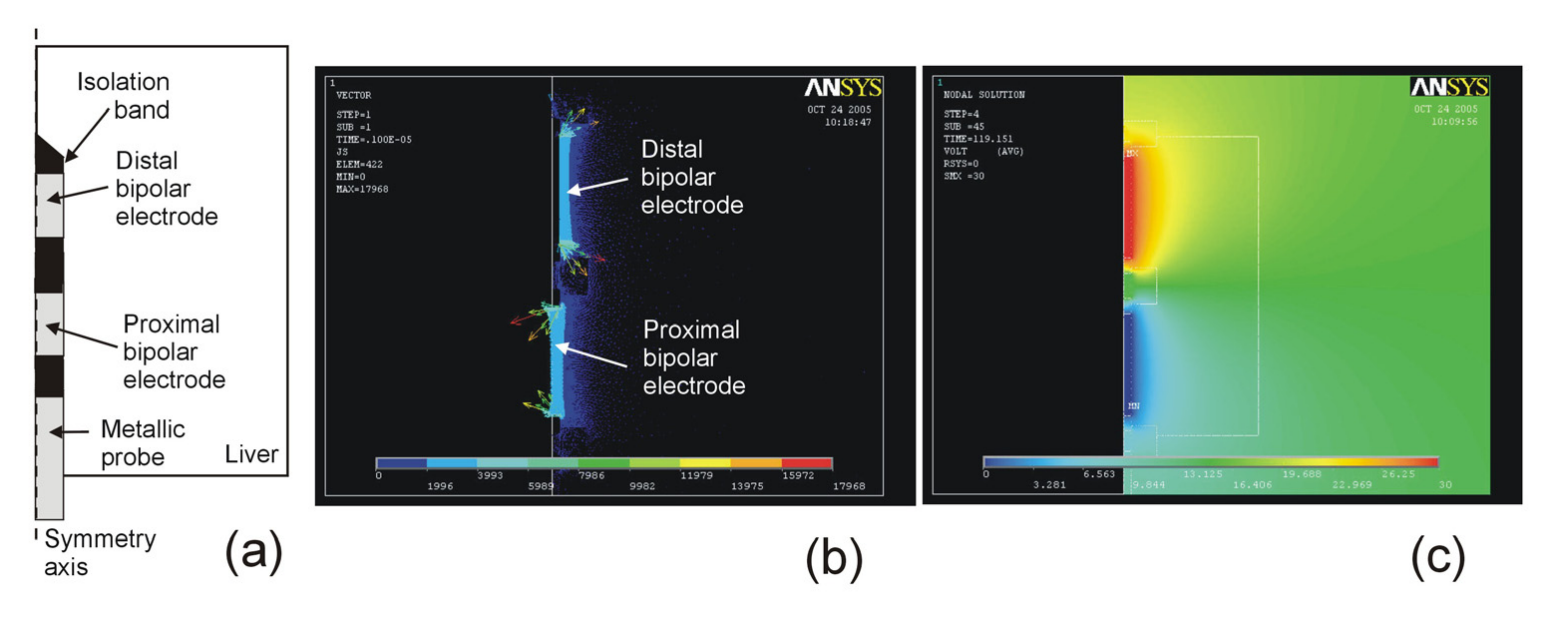
Example of post-processing of the theoretical modeling. (a) Two-dimensional model (axial symmetry) of a bipolar electrode for RF hepatic ablation similar to that proposed by Burdío et al [117]. (b) Current density (A/m^2^) distribution in the hepatic tissue surrounding the electrode. (c) Electrical potential (V) distribution.

The simplest RF ablation model is an electrical-thermal coupled problem. Therefore, the output variables are always electrical (voltage and current density) and thermal (temperature and heat flux). In some analyses, only electrical variables such as current density [27-29,34,39,55,71], electric field [36,48,55], and electrical potential [16] are employed (see Fig. 3). In fact, the spatial distribution of current density is directly related to the distribution of SAR (Specific Absorption Rate), which corresponds to the electrical power absorbed by the biological tissue [51,55,59]. On the other hand, other studies also calculated the total impedance (resistance) between active and dispersive electrode [17,23,25,27], particularly its time evolution, since this parameter is often registered by the RF generators [70]. The impedance value has also been used to model the quality of the contact between electrode and tissue prior to RF ablation [30,52]. Finally, some studies that modeled constant-power or constant-temperature ablation plotted the time evolution of the voltage applied on the active electrode [42,43,45,46,69].

Regarding thermal variables, temperature distribution is the most plotted result, due to its apparent association with thermal injury [14,17,23-25,27-29,32,34-38,40-45,48-55,59,60,62,63,69,71,72]. In other studies, it has also been interesting to plot the heat flux distribution [55].

Some modeling studies used an isothermal line to assess the tissue lesion boundary from the temperature distribution. Although different values have been used for this boundary, such as 48°C [25,62] or 59°C [44], 50°C was the most frequently employed [14,32-37,42,43,45,46,50,52,53,59,60,63,70] since it is known that the in vivo lesion volume (i.e. tissue rendered nonviable) after RF ablation can be defined by the volume enclosed by the 50°C isothermal surface [27]. However, the value of the boundary is not always chosen in order to estimate lesion dimensions, but rather to compare the computer results with the dimensions of changes macroscopically observed in tissue color after ex vivo or in vivo experiments. For instance, the discoloration observed in excised cardiac tissue when it is heated to 60°C, allowed in vitro results to be compared with computer simulations by considering the 60°C isothermal surface [27]. In another hepatic RF ablation study, the lesion border was determined by optical inspection, since the pale central area of the RF lesion corresponded to the necrosis zone (i.e. the 50°C isothermal surface) [36]. Once a boundary value has been chosen, it is then possible to estimate different lesion dimensions (depth, width, volume, or other specified diameters) [14,27,29,32,33,37,45,52,63,70] and their time evolution [23,42,43,46].

Nevertheless, since it is known that the biological damage is a function of both temperature and time, several authors have partially quantified it. Despite the fact that tissue damage can be associated with many different reactions, each with its own rate coefficient, it may be approximated in a single process [21]. As proposed by Henriques [94], this process is related to protein denaturation and can be characterized by a single rate constant of the Arrhenius form [95]. To be more precise, an arbitrary function of tissue injury (Ω) is defined as follows:



where *T *is the temperature (K) calculated at each point of the model region, *R *is the gas constant (8.3134 J/mole·K), *A *(s^-1^) is the frequency factor (a measure of molecular collisions) [96], and *ΔE *(J/mole) is an activation energy barrier which tissue constituents must surmount to denature [96]. Both *A *and *ΔE *are kinetic coefficients evaluated for each tissue type from experimental data, using both microscopic measurements (e.g. protein denaturation by means of scattering increase or birefringence loss) [88,94], and other more sophisticated techniques (e.g. expression of heat shock proteins (HSP) to track cellular response to thermal injury)[97,98]. At the same time, the values of the mentioned kinetics coefficients are chosen in order to make a value of Ω > 1 correspond to a tissue in which the thermal damage is completely irreversible [99].

So far, various theoretical models for RF ablation have employed this formulation to assess tissue damage, sometimes using skin data [94] due to the absence of specific data for the modeled tissue [23,24], or using specific data from previous experiments [54,96]. Other more complex models have been proposed, including not only cell death but also cell recovery [100]. More recently, a comparative study between the two methods of lesion assessment (50°C isotherm versus Arrhenius equation) for cardiac and hepatic RF ablation have concluded that the use of the isotherm could overestimate the lesion diameter of cardiac ablations with short treatment times (< 30 s), and underestimate it in hepatic ablations with long treatment times (> 20 minutes) [101].

## Experimental validation of the theoretical models

Once the theoretical models have been built, and although they are based on equations which correspond to well characterized phenomena, some type of experimental validation should be conducted to guarantee the results obtained from computer simulations. Many modeling studies have included experimental work that focused on the validation of theoretical models [22,24,27,29,31,33,35,36,38-40,44,45,48,49,54,59,70-72]. In these type of experiments, it is necessary to make a distinction between the material used as a heating target (model of biological tissue), the physical variables experimentally measured during, before and/or after the RF heating, and finally the experimental technique used to acquire these variables.

Firstly, concerning the material employed, the experiments can be conducted by following either of two methodologies: 1) using real biological tissue (previously excised -ex vivo or in vitro- [22,27,29,31,33,35,39,40,44,48,49,54,59,70,71], and in vivo [36,38,45,71,72]), and 2) using the so-called "phantom" tissue-equivalent-material [24,102-104], which is synthetic material with the same electrical and thermal characteristics as biological tissue. In this second case, special care should be taken to achieve these characteristics under suitable conditions of temperature (and frequency, in the case of electrical conductivity).

Secondly, the choice of the variables to be registered is strongly influenced by the availability and accuracy of the experimental techniques. For instance, it is obvious that the use of tissue-equivalent-material does not allow any subsequent histological analysis of the heated sample. However, it does allow, for instance, a number of temperature transducers, such as optic fibers [103], to be accurately placed or even to obtain temperature distributions on a transversal plane by means of a thermographic camera [24].

On the other hand, the use of real biological tissue offers other options for experimental validation. Some studies used temperature measurement at different locations in the tissue during the RF heating. This was mainly achieved by using several small transducers (e.g. thermocouples) placed with precision around the ablation zone [31,47,105]. In this respect, small temperature sensors such as thermistors have also been proposed for measuring SAR distribution in the tissue [106]. However in general, these procedure are not suitable for in vivo models, due to the practical difficulty of accurately placing the sensors. Additionally, RF electromagnetic fields induced in the tissue during RFA could cause errors affecting these thermometry techniques, and hence certain precautions have to be taken [107].

Since the use of small temperature sensors (thermocouples and thermistors) or thermographic image can have limitations in some cases, other experimental techniques have been proposed to obtain information on temperature distributions. For instance, Verdaasdonk and Borst [108] introduced a method based on Schlieren techniques, in which, using an optical setup, very small changes in optical density of the media induced by temperature gradients are color coded. To date, this methodology has been applied to study the thermal effects of lasers with high temporal and spatial resolution [109]. In the future, the application of RF ablation studies could contribute to better experimental validation of theoretical models.

Alternative methods of temperature measurement based on magnetic resonance imaging (MRI) have recently been employed for RFA of tumors in order to 1) interactively guide the RF electrode to the target, and 2) monitor the effect of therapy [110]. In fact, a modeling study of RF ablation of tumors [104] employed MRI to compare the lesion size during and post ablation to computer results. There are currently different techniques which show a correlation between the zone of irreversible tissue damage (i.e. the lesion dimension) and post-ablation MRI [111,112]. However, since RFA produces electromagnetic noise that may severely deteriorate MR image quality, new techniques such as thermosensitive MRI contrast agents are being experimentally tested. These agents show a change of state (from MR-inactived to MR-actived) when temperature increases from physiological temperature to a phase transition temperature [113]. It is very possible that experimental validation of theoretical models will make use of all these techniques in the not so distant future.

**Figure 4:**
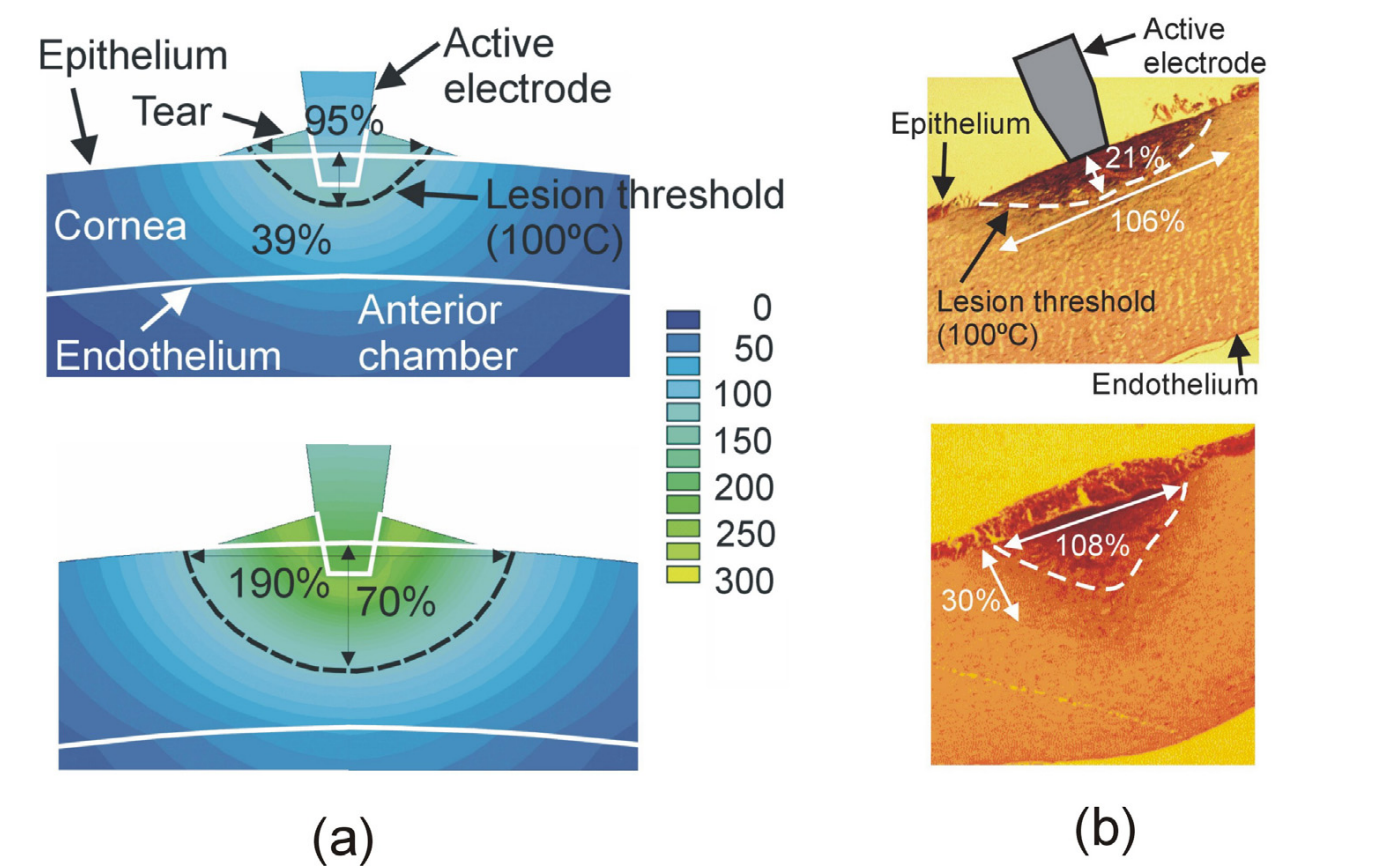
Example of experimental validation of the theoretical models by comparing computed temperature distributions and histologic samples. (a) Temperature distributions in the cornea during RF heating using constant voltage (scale in °C). Top: 16 V after 1 s. Bottom: 21 V after 1 s. (b) Cross-section of corneas heated with an active electrode of 200 μm diameter (hematoxylin and eosin, ×40). Top: 16 V after 1 s. Bottom: 21 V after 1 s. The lesion dimensions (central depth and surface width) were assessed using the coagulation contour, and were expressed as % of cornea thickness. In this case, the 100°C isothermal line was used to compare the experimental and computed results since it had been observed that the coagulation contour occurs in the temperature range around 100°C using short heating times [49].

When temperature measurement was not possible or appropriate, some studies compared the computed temperature distributions (or the line of irreversible damage computed from a thermal injury function) to the macroscopic and/or histological samples of the heated tissue. For example, the macroscopic assessment of cardiac tissue was based on the degree of discoloration in the lesion zone [22,71], which corresponds to a temperature of 60°C [27]. Since this isothermal line underestimates the real zone of permanently damaged tissue [114], some studies have stained the heated tissue sample with a special solution in order to better distinguish the geometry of nonviable tissue [29,31]. In other cases, when an in vivo model was used to perform the experimental validation, the lesions were analyzed and two visible zones were identified: a well-demarcated necrosis zone, and a surrounding zone of hemorrhage and inflamed cells. In this case the outer boundary enclosing both zones was considered as the lesion border and hence compared to the 50°C isotherm of the computed temperature distributions [45]. Likewise, for hepatic tissue, the validation procedures have included both the analysis of the tissue discoloration (pale zone) [35,36], and the use of special solutions for staining the tissue [54]. Alternatively, mainly due to limitations in size, some studies have proposed the use of histologic analyses (see Fig. 4) [48,49,59]. Future research on the histologic signatures of thermal injury will allow a more accurate comparison to be made between computer and experimental results in this field [115].

Finally, some studies have considered the basal value and/or the time evolution of electrical variables during the heating in order to compare the computed and experimental values. Since the total impedance between the active and dispersive electrodes decreases during heating, the evolution of this parameter has occasionally been employed to experimentally validate theoretical models [17,24,116].

## Current limitations

### Modeling saline-enhanced RF ablation

Some RF hepatic ablation procedures involve the use of a simultaneous saline infusion in the tissue [40,117,118]. In this case, the theoretical model becomes extremely complicated, since there is not only an electrical-thermal coupled problem, but also a fluid dynamics problem in a porous medium (with preferential paths through the principal veins). Since this fluid is usually a hypertonic saline (with a high value of electrical conductivity) the spatial distribution of the fluid in the tissue has a significant effect on the electrical problem (i.e. the electrical energy distribution). Also, since the fluid is infused during heating using a significant rate at ambient temperature, two thermal effects are produced which are at present difficult to include in a model: 1) a cooling effect in the region of the electrode, and 2) a convection effect due to the fluid being heated in the proximity of the electrode (once infused through the outlets). The fluid then moves away from the electrode and heats remote tissues. Finally, these procedures usually involve temperature values approaching 100°C, which implies a clearly non linear behavior of the tissues. Accurate modeling of saline-enhanced RF ablation is consequently not feasible at the present time.

### Modeling of the power delivery protocol

RF ablation procedures are conducted using an electrical generator very similar to those normally employed in electrosurgical practice. This generator can operate in different modes such as pulsed [119] versus continuous wave, damped [17] or non-damped pulses, and constant current (high output impedance) versus constant voltage (low output impedance). Most modeling studies have considered only constant voltage, power and temperature modes, and only a few modeled pulsed voltage [17,24].

On the other hand, no theoretical models have been proposed that include the effect of the impedance output of the RF generator, i.e. the electrical boundary conditions used in the active electrode implicitly considered as ideal electrical sources. This issue could be significant since the current RF generators present output impedances which could be similar to the value of the load impedance (tissue impedance). This means that during an actual RF heating, the resulting decrease in the tissue impedance could cause a mismatching of the two impedances, and hence significant errors in the computer results (considering an ideal voltage source).

Another interesting question is the modeling of the control algorithm employed in RF generators that use constant temperature. Recently, Haemmerich and Webster [69] have implemented a PI (Proportional-Integral) controller for RF ablation models. However, since this is not the only algorithm available for commercial RF generators, it is also necessary to carry out more research into the characteristics of the control loops usually employed in these generators in order to include them in future modeling studies.

### Characterization of biological tissues

Even though great efforts have been made to obtain an accurate value for each of the characteristics of different biological tissues, it is important to take two issues into account. On one hand, the dispersion of the values of the biological characteristics can become very important, due to the variability between individual values, and the changing environmental and physiological conditions. Some modeling studies have assessed the impact of these changes on temperature distributions considering increments and/or reductions of up to 100% [14,49,70,80].

On the other hand, to date, theoretical RF ablation models and their corresponding computer simulations have only been related to the comparative thermal dosimetry [13]. The aim of this tool is, from a very general point of view, the comparative evaluation of the potentials of different heating modes and configurations. Specifically, the models developed have made a comparative assessment of the effect of: geometry [27,28], dimensions [23,25,29,48], and arrangement [34,36,39] of the active electrode(s), the type of ablation (monopolar versus bipolar)[16,36], the insertion depth of the electrode into the tissue [29,32,46,52], the value of applied voltage [23,55], the condition of thermal cooling around the active electrode [27,31,32,44,60] and in the interior of the electrode (cooled electrode) [35,41,68], the protocol for delivering RF energy (constant voltage, constant power or constant temperature) [42,43,69], the thickness of the ablated tissue [51,52], the presence or lack of adjacent significant structures, such as adipose layer [51,52], blood vessels [34,37], or tumor [59], the location of the dispersive electrode [25,45], the tissue perfusion [54], and the temperature and flow of the saline infused during the RF ablation [40]. In all these studies, standardized models were used, i.e. only the most significant anatomical and physiological features of "typical" patients were considered [13]. In other words, the results obtained from modeling studies should be considered qualitatively rather than quantitatively [60]. In fact, the sensitivity analyses demonstrate that, even when the precise value of the tissue characteristics is unknown, the conclusions of comparative studies are accurate, since the electrical-thermal behavior remains constant even when the values of the characteristics vary. This consideration is not only valid for the tissue characteristics, but also for any anatomic or physiological datum.

In conclusion, it does not seem either important or urgent to obtain the precise characteristics of each type of biological tissue. However, it is urgent and necessary to know the relationship between tissue characteristics and temperature, in order to accurately model certain RF heating techniques. In fact, as was stated at the end of the section "Physical characteristics of biological tissues", there is at present a considerable lack of understanding of the changes in the physical characteristics of biological tissues during intense heating, i.e. when temperature reaches ≈100°C. In these conditions, it seems obvious that all the characteristics will experience sizeable, and probably irreversible, changes in value. It is therefore both urgent and important to conduct experimental studies to assess these behaviors. This is especially necessary in the modeling of RF ablation procedures in which very high temperatures are reached, such as hepatic RF ablation using saline irrigation [117,118] or RF thermokeratoplasty [17].

## Benefits of the methodology

The computer modeling of RF ablation offers several unquestionable advantages over the experimental approach. For this reason, it has become an essential tool to complement experimental studies on RF ablation techniques. Not only is it less expensive and faster than ex vivo and in vivo experiments, but it also allows the time evolution and spatial distribution of physical variables to be analyzed. These values are impossible to monitor due to the lack of suitable transducers. These advantages will provide inestimable help to the research and development processes of the manufacturers of RF ablation systems. This is an important advantage, but in addition, and as I have gathered from my experience of cooperation with surgeons, radiologists, and cardiologists, RF ablation models offer valuable assistance in explaining the biophysical phenomena involved in the RF heating of biological tissues. In other words, the models are excellent didactic tools that enable the users of RF ablation systems to become familiar with the equipment and procedures, and thus indirectly enhance the safety and efficacy of the therapies.

A number of studies have recently proposed that theoretical modeling might be useful not only as a support in the design and understanding of the phenomenon, but also to provide guidance during the ablation procedure. For instance, various models have been developed for predicting lesion size during catheter cardiac ablation using previous information (e.g. location of the ablation in the cardiac chamber, insertion depth of the electrode in the tissue, and preset temperature) [32,33]. Concerning RFA of tumors, it has been proposed that theoretical models and fast computer simulations be simultaneously combined with MRI to predict tissue temperature during a procedure, thus increasing the effectiveness and reliability of the ablation [58]. In fact, MRI has already been employed to assess the computer results of theoretical modeling [104]. In conclusion, these applications would allow RFA computer modeling to be used as a tool for quantitatively planning the thermal dose in individual cases.

Finally, although all the foregoing is related to radiofrequency ablation, the methodology described is very similar to those employed to study other thermal techniques for destroying biological tissues. In fact, numerous computer modeling studies have also been published on techniques such as laser-induced interstitial thermotherapy (LITT) [120-122], high intensity focused ultrasound (HIFU) [123-125], microwave ablation [126-129], cryoablation [130,131] and thermal balloon endometrial ablation [132,133].

## Research objectives for the near future

**Figure 5:**
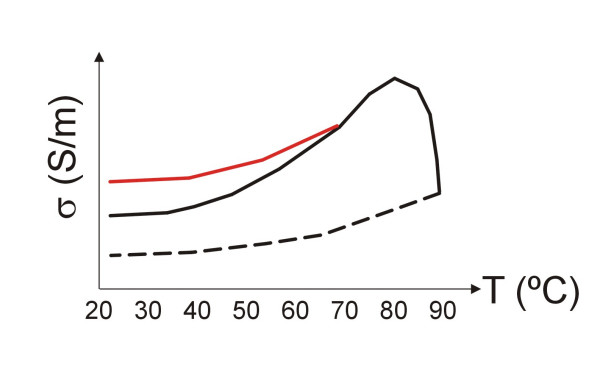
Example of hypothetic behavior for the electrical conductivity (*σ*) of a biological tissue considering a thermal level of ≈70°C as first threshold of the irreversible behavior, such as that experimentally observed by Pop et al [82]. In addition, the dotted line represents the relation between *σ *and temperature once a second thermal threshold (≈90°C) has occurred. This second level is associated with a high degree of tissue desiccation, and thus a significant increase in the electrical impedance, as has been experimentally observed [134] (i.e. a lower value of *σ*). The real situation is probably more complex, since the thresholds are not simply thermal levels but levels of a tissue damage function. For the y-axis the units of *σ *are arbitrary.

The future of theoretical RF ablation modeling appears to lie in:

1) *Accurate modeling of the electrical and thermal characteristics of biological tissues*, not only those that are temperature-dependent, but also time-dependent, i.e. to quantify the relations between the values of the characteristics and the thermal damage function. In addition, these relations offer irreversible effects above a certain thermal level (≈70–80°C), or over a specific value of a thermal damage function [82,83]. This fact would imply a hysteresis in the relation between the tissue characteristics and temperature, or temperature-time. Fig. 5 shows, merely as an illustration, an example of this behavior for the electrical conductivity (*σ*) of a biological tissue considering a thermal level of ≈70°C as the threshold of irreversible behavior (red line). This behavior has been experimentally assessed. However, it is known that a tissue temperature value of ≈90°C is associated with a high degree of tissue desiccation, and thus to a significant increase in electrical impedance [134] (i.e. a lower value of *σ*). Thus, it seems reasonable to consider a second thermal threshold (probably around 90–100°C) which involves a more or less abrupt drop in *σ *(see Fig. 5). In addition, once this second threshold has been exceeded, the relationship between *σ *and temperature might follow a curve such as that suggested by the dotted line (i.e. the tissue remains more or less desiccated even after cooling). All these relations should be studied in future experimental work.

2) *Realistic modeling of the cooling effect of large and medium blood vessels*. This issue is especially important in RF ablation of tumors, in which the thermal cooling effect of tissue perfusion may dramatically reduce the lesion size [135], and the proximity of large vessels [136] may imply lesions with irregular shapes which are not matched to the tumor volume [137]. As a consequence, the ablation might not be able to completely destroy the tumor. A number of modeling studies have considered the presence of blood vessels [34,37] and assessed their impact on temperature distribution. Experimental studies were recently conducted to quantify the thermal effect of the blood circulating in arteries and veins [138,139] and in the endocardium [140]. The data obtained from these and future experimental studies could be employed in a computer model. A comprehensive review of the thermal modeling of blood vessels and its influence on the Bio-heat equation the can be found in [99].

*3) Determining the parameters (frequency factor and energy) of the thermal damage function for different types of tissues (hepatic, breast, cardiac, etc.)*. For this purpose, it is possible to use the classical methods (such as the measurement of the decrease in intensity of the thermally induced birefringence image using polarizing microscopy [99]), or to employ new techniques to improve the understanding of the lethal and sublethal injury on a cellular level [97]. Finally, the value of the damage function could be used to modify the value of various terms in the Bio-heat equation, such as the heat loss by local tissue perfusion [54].

*4) Conducting research on new histological markers of thermal injury *to allow consistent experimental validation using ex vivo and in vivo samples. These markers would allow different histological changes to different isothermal lines to be compared [99,115,141,142].

*5) Development of fast computer simulation of ablation models *to predict tissue temperature and hence to provide simultaneous guidance during a procedure [58,143]. In this respect, and with the support provided by the new methodologies of planning, simulation, and training [143,144], theoretical modeling could become a tool for quantitatively planning individual treatment by using MRI or other future techniques.

6) Finally, it is especially important *to obtain a more accurate model of the behavior of the tissue during the simultaneous application of RF energy and saline perfusion*. This is a truly complex phenomenon and to date only a one-dimensional model has been developed [40]. In fact, once the saline is infused into the tissue, it produces a cooling effect in the proximity of the electrode [40]. The saline is simultaneously heated by the effect of the SAR, and probably pushed towards deeper zones in the tissue, thus enlarging the lesion size by thermal convection, in addition to conduction. As a result, the modeling problem turns out to be very complex, since several areas of physics are involved (heat transfer, electronics and fluid dynamics [60]).

On the other hand, it seems that certain other lines of research are of low priority, due to their currently high cost in human resources and computational power, as well as to an apparent lack of utility, as is the case, for instance, of large-scale modeling including an entire human torso to study RF cardiac and hepatic ablation. Another questionable issue would be the inclusion of an extremely realistic geometry in the models, since the key question of any model is its simplicity, and only the genuinely significant aspects should be included.

## Conclusion

Radiofrequency ablation (RFA) is a surgical technique that in recent years has come to be employed in very diverse medical fields. In order to study, investigate and develop new techniques and to improve those currently employed, research can make use of clinical and experimental studies, phantoms, and theoretical models. The latter are a powerful tool in this kind of investigation, since they rapidly and economically provide an understanding of the electrical and thermal behavior involved in ablation. In the last 10 years several groups have developed theoretical models for the study of RF ablation. In this review, the methodology of the modeling has been explained, including the experimental validation. At present, certain important limitations impede the complete and accurate development of the model, especially under conditions of high temperature (≈100°C) or simultaneous saline perfusion. In spite of this, modeling has grown to such an extent that it has become an essential tool in assisting experimental studies on RF ablation techniques.
